# Novel Insights into the Molecular Mechanisms of Atherosclerosis

**DOI:** 10.3390/ijms241713434

**Published:** 2023-08-30

**Authors:** Armanda Wojtasińska, Weronika Frąk, Wiktoria Lisińska, Natalia Sapeda, Ewelina Młynarska, Jacek Rysz, Beata Franczyk

**Affiliations:** 1 Department of Nephrocardiology, Medical University of Lodz, ul. Zeromskiego 113, 90-549 Lodz, Polandlisinskawiktoria324@gmail.com (W.L.);; 2 Department of Nephrology, Hypertension and Family Medicine, Medical University of Lodz, ul. Zeromskiego 113, 90-549 Lodz, Poland

**Keywords:** atherosclerosis, cardiovascular diseases, inflammation, endothelial dysfunction, uric acid, vitamin D, miRNA expression, molecular mechanisms

## Abstract

Atherosclerosis is one of the most fatal diseases in the world. The associated thickening of the arterial wall and its background and consequences make it a very composite disease entity with many mechanisms that lead to its creation. It is an active process, and scientists from various branches are engaged in research, including molecular biologists, cardiologists, and immunologists. This review summarizes the available information on the pathophysiological implications of atherosclerosis, focusing on endothelium dysfunction, inflammatory factors, aging, and uric acid, vitamin D, and miRNA expression as recent evidence of interactions of the molecular and cellular elements. Analyzing new discoveries for the underlying causes of this condition assists the general research to improve understanding of the mechanism of pathophysiology and thus prevention of cardiovascular diseases.

## 1. Introduction

Cardiovascular disease (CVD) is the biggest cause of mortality in the world [1,2]. Atherosclerosis is the leading cause of mortality from CVD globally. It is a thickening and hardening of the artery wall that occurs with aging and is linked to profound consequences for the cardiovascular system and multiple other disorders. The identification of atherogenesis as an active process rather than a passive cholesterol storage disease has highlighted important inflammatory, molecular, and cellular pathways [3]. Inflammation, aging, endothelial dysfunction, uric acid (UA), the impact of vitamin D (vit. D), and miRNA were discussed in our previous article [4]. UA has been linked to arterial calcification and thickening of the intima, which causes vascular calcification and hastens atherosclerosis [5]. Calcitriol (1,25(OH)2D) is the primary cause of vit. D’s systemic effects, including anti-inflammatory, antithrombotic, and anti-atherosclerotic properties [6]. MicroRNA (miRNA) regulates the expression of genes, which therefore affects the level of synthesized protein in cells which in turn participates in atherogenesis. In this review, we will summarize the existing information on atherosclerosis, with an emphasis on clarifying the mechanism behind its creation and evolution.

### Atherosclerosis

Recent observations established that atherosclerosis is the leading cause of vascular disease globally [7,8]. According to the Progression of Early Subclinical Atherosclerosis study, middle-aged men and women suffer from subclinical atherosclerosis in 71% and 43% of cases, respectively [9]. Atherosclerotic cardiovascular disease is a major factor in morbidity and mortality in the Canadian and Swedish populations [10,11,12]. An estimated 17.9 million people died from CVD in 2019, representing 32% of all global deaths. Of these deaths, 85% were due to a heart attack or a stroke [12]. Dominantly, atherosclerosis is the underlying cause of the majority of CVD cases [13].

Atherosclerosis is considered to be caused by multiple factors, including genetic and environmental factors. There are many known risk factors for atherosclerosis, including hypercholesterolemia, hypertension, diabetes mellitus, kidney diseases, and cigarette smoking [14].

Symptoms of CVD have a significant impact on one’s quality of life. They are examined as patient-reported outcomes in terms of their genesis, expression, similarities, and differences between illnesses. Atherosclerosis could be latent and symptomless, but the consequences are quite diverse. The symptoms of acute coronary syndrome (ACS), heart failure, valve problems, stroke, rhythm abnormalities, and peripheral vascular disease are considered to be the most common consequences of atherosclerosis [15]. Chest discomfort is the most frequently reported ACS symptom. Substernal pressure or discomfort is a common description of the chest pain, which can radiate to the mouth, shoulder, arm, or upper back. Dyspnea, diaphoresis, unusual weariness, nausea, and light-headedness are the most typical symptoms co-occurring with chest discomfort [16]. The most common symptom of heart failure is dyspnea (also known as shortness of breath, breathing pain, or breathlessness) [17]. Dyspnea is frequently classified by provocation, which includes dyspnea at rest, dyspnea during exertion, orthopnea, paroxysmal nocturnal dyspnea, and bendopnea [18]. In the case of acute stroke syndrome, there is an acronym which describes the main symptoms: facial, arm, speech, and time (FAST). Associated abbreviations were created to aid the lay public’s recognition and prehospital reaction to the most prevalent stroke symptoms. These can include weakness and numbness, speech difficulties, disorientation, dizziness, loss of coordination or balance, and visual abnormalities [19].

This chronic disease can escalate for many years and can lead to serious complications due to ongoing inflammatory processes. Atherosclerosis results in CVD, which can take the form of ischemic heart disease, stroke, or other vascular diseases [20].

Moreover, chronic kidney disease (CKD) is also a complication of atherosclerosis. The composition of atherosclerotic plaques is responsible for the severity and advancement of CKD. For this very reason, it is necessary to start treatment at an early stage of atherosclerosis to achieve the best results [21].

The pathogeneses of atherosclerosis and diabetes are closely related. Diabetes has been shown to be a triggering factor for atherosclerosis due to dyslipidemia, hyperglycemia, oxidative stress, and chronic inflammation. These components are common in both diseases [22].

The most crucial recommendations for the prevention of atherosclerosis include a proper diet, physical exercise, smoking cessation, adequate stress management, and good quality of sleep [23].

Meat consumption, especially that of red meat and processed meat products, should be limited to 0.5 kg per week [24,25]. Fish [26], legumes, and eggs [27] are recommended (One egg per day does not increase plasma low-density lipoprotein (LDL) cholesterol). Fatty fish meat is a source of unsaturated ω-3 fatty acids. Legume seeds provide valuable protein, fiber, and vitamins B1, B6, and PP. Meals should be eaten regularly [28]. Vegetables and fruits should be eaten as often and as much as possible (≥400 g/d). They are a source of a large amount of bioactive compounds (carotenoids, vitamins C and E, folic acid, selenium, flavonoids, and isoflavones), potassium, and fiber [29]. Cereal products play a vital role in the diet. Whole grain products provide B vitamins, minerals (magnesium and zinc), and dietary fiber. The carbohydrates in them are a source of energy [30]. Lean dairy products are recommended [31]. Consumption of animal fats (saturated fatty acids) should be replaced with non-tropical vegetable oils (unsaturated fatty acids) [32]. Consumption of sugar and sweets should be limited since they contribute to the development of obesity, being overweight, and consequently, type 2 diabetes and elevated plasma triglycerides [33]. Salt intake should be limited [34]. It is recommended to not consume alcohol [35].

As part of the prevention of cardiovascular diseases (CVDs), healthy people are recommended to undertake physical activity, namely either over 150 min/week of moderate intensity aerobic exercise, 75 min/week of high-intensity aerobic exercise, or an equivalent to either of these [36].

## 2. Inflammation

**Figure 1:**
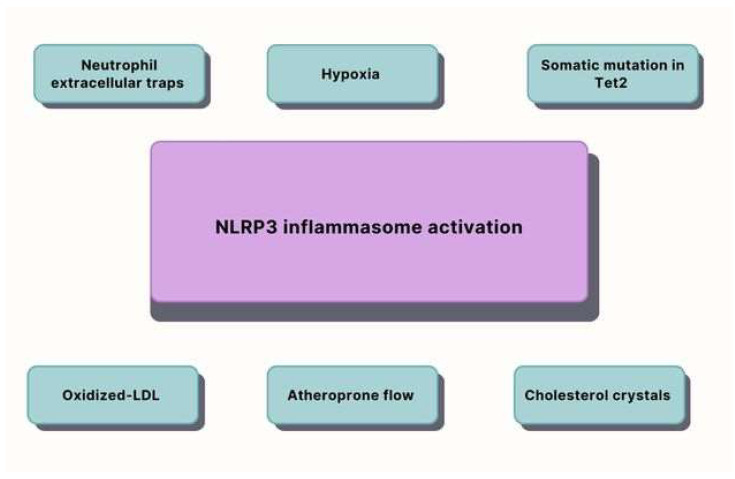
The graph shows key factors that participate in NLRP3 inflammasome activation, which is the onset of inflammation in atherosclerosis. Tet2 = ten-eleven translocation 2; NLRP3 = nucleotide-binding domain, leucine rich containing family, pyrin domain containing 3; and Oxidized-LDL = oxidized low-density lipoprotein.

Atherosclerosis is chronic arterial inflammation caused by both conventional [37] and unconventional risk factors that result in plaque development in the vascular intima. In the past, atherosclerosis was thought to be a condition brought on by the retention of lipoproteins, particularly low-density lipoprotein (LDL), in the inner lining of arteries. Russell Ross originally proposed the idea that atherosclerosis is an inflammatory disease in 1999, based on his observations that circulating monocytes enter the growing fatty streak. Intimal infiltration, modification of plasma-derived lipoproteins, and their primary uptake by macrophages result in lipid-filled foam cell formation. This is the onset of atherosclerotic lesions. Inflammation starts with inflammasomes, which are innate immunological signaling complexes that are a significant modulator of IL-1 family cytokine production in atherosclerosis, contributing to the vascular inflammatory response that drives the development and progression of atherosclerosis [38]. An inflammasome is a protein complex that detects external danger signals and releases activated cytokines such as IL-1 family cytokine. It is the main factor in vascular inflammation and plays a fundamental role in the initiation and progression of atherogenesis. The main components of the best-demonstrated NLRP3 and NLRP1 inflammasomes include the adaptor apoptosis-associated speck-like protein containing a zymogen pro-caspase-1 and an NLR family member [39]. These components of the NLRP3 inflammasome are dominantly elevated in macrophages and foam cells within human carotid atherosclerotic plaques (including activated caspase-1) in comparison with their healthy counterparts [40,41]. The major factors that activate the NLRP3 inflammasome in atherosclerosis are shown in Figure 1. Hypoxia dominates as a cause of atherosclerotic lesions, promotes plaque angiogenesis and foam cell formation, and participates in formation of the plaque’s necrotic core. Because of excessive oxygen consumption and diffusion constraints in the plaque, atherosclerotic plaques feature hypoxic zones. Although severe hypoxia predominates in deep atherosclerotic plaque sections, most cells including macrophages in rupture-prone regions undergo persistent mild hypoxia (2–5% O^2^) [42]. This low oxygen level promotes plaque angiogenesis, which can lead to lesion progression, intraplaque bleeding, oxidative stress, and inflammatory cell recruitment. Hypoxia promotes foam cell production in mononuclear phagocytes by increasing fatty acid synthesis and blocking fatty acid oxidation and cholesterol exportation. Hypoxic cells switch from mitochondrial respiration to anaerobic glycolysis, resulting in the production of reactive oxygen species and decreased adenosine triphosphate (ATP) availability, conditions that predispose to cell death and contribute to formation of the necrotic core of the plaque [43]. Furthermore, hypoxia not only increases the NLRP3 activation in macrophages and plaques but also pro-IL-1 protein expression with exclusion of mRNA expression in LPS-stimulated human macrophages. Hypoxia also prevents pro-IL-1 from being selectively targeted for autophagic destruction, extending its half-life and boosting intracellular accumulation. Moreover, hypoxia enhanced the expression of NLRP3, which is a limiting factor in the activity of the NLRP3 inflammasome, and increased caspase-1 activation in LPS-primed macrophages. As a result, after treatment with crystalline cholesterol, hypoxia human macrophages produced more mature IL-1 than normoxic macrophages, an endogenous danger signal that contributes to atherogenesis [44].

Afterward, the active NLRP3 inflammasome triggers the recruitment and activation of caspase-1, which participates not only in the activation of the precursors of IL-1β and IL-18 but also in the breaking of gasdermin D [45]. IL-1β and IL-18 are released and recruit neutrophils. Neutrophil extracellular traps (NETs) are web-like DNA structures made up of cell-free DNA, histones, and neutrophil granule proteins [46]. In response to infections and inflammatory stimuli such as cholesterol crystals, oxidized low-density lipoprotein, oxysterols, platelets, and chemokines, neutrophils release cytosolic and nuclear material, generating a net-like extracellular structure [47]. Microbicidal nuclear and granule proteins bind to these NETs, encouraging their eradication. Uncontrolled NET production during inflammation, on the other hand, can produce vascular blockages, tissue damage, and protracted inflammation, worsening a variety of pathological diseases such as atherosclerosis [48]. NETosis can also induce inflammation by boosting monocyte recruitment and the activation of macrophages to generate reactive oxygen species and proinflammatory cytokines [49]. Neutrophil extracellular traps (NETs) can induce activation of the NLRP3 inflammasome in macrophage cytokine release, activating the Th-17 cells that amplify immune cell recruitment in atherosclerotic plaques. The NLRP3 inflammasome also plays a crucial role in mediating ten-eleven translocation 2 (Tet2) mutation, which participate in onset of atherosclerosis, which is further explained in the following section on aging. Excessive production of inflammatory cells under hypercholesterolemic conditions has a causal role in promoting atherosclerotic CVD. CCs, the major drivers of atherosclerosis, are now considered the most important trigger for NLRP3 inflammasome activation [41]. This phenomenon is undesirable since it can lead to lipid peroxidation and disrupt the physiological functioning of bio enzymes. Oxidized phospholipids are known to cause inflammation. For this reason, oxidized low-density lipoproteins (oxLDLs) are referred to as a clinical marker of plaque inflammation [50]. From the mechanisms of endothelial dysfunction, it can be observed that inflammatory variables also have a significant impact on the pathogenesis of this disorder. The intima of a damaged artery wall contains lymphocytes and mast cells [51]. Modified lipids trigger the intima’s inflammatory cells to produce chemokines and cytokines like tumor necrosis factor α (TNFα), interleukin-1, -4, and -6, and interferon-gamma. These chemokines and cytokines then trigger the activation of other leukocytes, endothelial cells, and adhesion molecules, particularly vascular cell adhesion molecule-1 (VCAM-1). Atherosclerotic plaques are created from these changed lipoproteins [52]. The cholesterol-rich lipoproteins from LDL are absorbed and incorporated into macrophages, and they also emit ROS and reactive nitrogen species (RNS), which are pro-oxidants that aid in the development of atherosclerosis. Increased levels of ROS may be caused by an impaired mitochondrial function, which is related to aging [53]. Endothelial cells are agitated by the oxLDLs, which increases the creation of molecules that form adhesions. The LDL-cholesterol (LDL-C) is converted by the ROS and RNS into ox-LDLs, which are a component of the intimal layer. Finally, an atherosclerotic plaque is formed with components of fiber tissue, muscle cells, and inflammatory cells. Accelerated cell turnover is anticipated to increase macromolecular permeability, enhancing lipid absorption in disturbed flow areas. This, in turn, would increase the atherosclerotic phenotypic expression of the vascular endothelial growth factor in response to modest shear stress, resulting in increased endothelial permeability [50].

Inflammation starts with the activation of NLRP3 inflammasomes, which results in the production of proinflammatory cytokines IL-1 and IL-18, acting via the autocrine, paracrine, or endocrine pathways [54]. IL-1 has been demonstrated to promote its own gene expression in a variety of cell types through an amplification loop known as autoinduction. IL-1 increases endothelial dysfunction, leukocyte-endothelial cell adhesion, procoagulant activity, and neutrophil recruitment, all of which contribute to atherogenesis and plaque ruptures [55]. Interaction of the IL-1, IL-1β, and IL-18 with their respective receptors in the extracellular space results in the activation of reactive oxygen species (ROS), matrix-degrading enzymes, T-cell activation and proliferation, and further cytokine production [56]. Hence, the result of NLRP3 activation is IL-1 and IL-18. The CANTOS trial found that canakinumab, which is a human monoclonal antibody, can antagonize the interaction between interleukin-1β and interleukin-1R. In effect, major adverse cardiovascular events are reduced, including myocardial infarction, stroke, cardiovascular death, and plasma inflammatory markers without affecting LDL cholesterol or HDL cholesterol levels.

## 3. Aging

Aging is one of the strongest risk factors for atherosclerosis [55] which increases the morbidity and mortality of patients. Understanding the mechanisms of the age-related increase in atherosclerotic diseases can better guide prevention and therapy in this risk group, since it is unclear whether aging itself increases the susceptibility to atherosclerotic diseases and their severity [57]. In this review, we present two main areas in which aging promotes atherosclerosis. The first group of factors is those outside of the vascular system, such as the impact of age on the clonal hematopoiesis of indeterminate potential (CHIP) differentiation of hematopoietic cells in the myeloid cell lineage and advocacy of the development of clones without obvious clonal disorders or hematopoietic malignancies. CHIP is strongly associated with mutations in Tet2, which are also seen in age-related cardiac hypertrophy and fibrosis [58]. Multiple studies have proven that the deletion of one copy of the Tet2 gene is adequate to an increase atherosclerosis occurrence in mice [59,60,61]. Moreover, myeloid cell-specific Tet2 deficiency increases the atherosclerotic plaque size [62]. Furthermore, a Tet2 deficiency in bone marrow-derived macrophages results in elevated secretion of IL-6 and IL-1. The level of these cytokines is also mediated by the vascular smooth muscle cells (VSMCs), mitochondrial genomic instability, and a decline in mitochondrial function. Aging participates in the production of chemoattractants, which increase myeloid cell recruitment into the arterial wall, further promoting atherosclerosis. The presence of CHIP increases the chances of CVD, but a loss of function mutation in IL-6 reduces this effect [53,63]. The second group of factors is the vascular intrinsic ones, such as the effect of aging on vascular bioenergetics due to impairment of mitochondrial function, mitophagy (removal of damaged mitochondria), and an impact on inflammation in vessels [63]. In addition, mtDNA damage, which is an early event of atherogenesis in apolipoprotein deficient (ApoE) mice, can result in mitochondrial dysfunction, leading to proatherogenic processes such as inflammation and apoptosis. Endogenous mtDNA damage in atherosclerosis in mice and humans decreases the mitochondrial copy number and respiration but promotes the proliferation of human VSMCs and macrophage apoptosis. This leads to a reduction in the necrotic core and an increase in fibrous cap areas [64]. Moreover, ox-LDLs can induce mitophagy in VSMCs [65]. Aging increases IL-6 secretion and impairs mitochondrial function in the aorta, which is associated with increased mitophagy and elevated parkin levels. The IL-6 in the aorta occurs in a positive feedback loop with vascular mitochondrial dysfunction. These age-related changes cause the vasculature to exacerbate atherogenesis in acute hyperlipidemia [55]. Another important factor related to aging is the proprotein convertase subtilisin/kexin type 9 (PCSK9). PCSK9 not only promotes the breakdown of hepatic low-density lipoprotein receptors (LDLRs) and thereby decreases LDL-C uptake in hepatocytes but also increases hepatic lipid and lipoprotein production via ApoE- and LDLR-dependent mechanisms. However, human PCSK9 accumulates in the arterial wall and directly influences the size and composition of the atherosclerotic lesion. These effects of human PCSK9 depend on LDLR but are independent of ApoE [66]. Different studies tried to determine the influence of PCSK9 on spontaneous and oscillating shear-induced atherosclerotic lesions in two adheropronous mouse models (the ApoE mouse and the C57BL/6 mouse) treated with an adeno-associated virus (AAV) to upregulate PCSK9, which controls LDL receptor expression at the plasma membrane. As a result, it has an impact on the LDL level in the blood [67]. This study assessed plaque loading of the aorta, the size and morphologic grade of the atheromas in the aortic root, and the carotid artery after oscillatory shear stress induction by partial carotid ligation (PCL). These measurements were performed on young, middle-aged, and old ApoE mice and on young and old PCSK9-overexpressing C57BL/6 mice, bothof which were fed an atherogenic diet. The results of this study were the following:

1. Age increases the aortic plaque burden and the size and severity of the aortic root plaques in the AD-fed ApoE mice independent of the number of weeks on the diet. However, there was no effect from age on the size or severity of oscillatory shear-induced carotid artery atheromas after PCL.
2. Treatment with PCSK9 increased the total and LDL cholesterol in the young mice, but the older mice had a larger aortic root atheroma size and morphology grade. Age did not affect the size or degree of the carotid artery lesions in the mice overexpressing PCK9 after PCL. Atherosclerotic plaques will expand spontaneously in the atheroprone regions of the aorta of the AD-fed mice after PCSK9 overexpression by an AAV. The transgenic ApoE mice also overexpressed human PCSK9, demonstrating larger atherosclerotic lesions with greater monocyte infiltration compared with the PCSK9 wildtype ApoE mice [57].

## 4. Endothelial Dysfunction

The vascular endothelium, as an integral component of the cardiovascular system intimately interfacing with the blood, plays a crucial role in maintaining systemic homeostasis. It acts as a lining of the cardiovascular system, forming a particular barrier to various molecules [68,69,70]. It assumes multifaceted functions within the human body, being intricately responsive to a myriad of external stimuli present in the surrounding environment [52,69,70,71]. The vascular endothelium plays a regulatory role in vascular muscle contraction, relaxation, smooth muscle proliferation, and the expression of adhesion molecules or chemotactic factors. These factors are responsible for the adhesion, activation, or migration of inflammatory cells, as well as platelet adhesion and aggregation. Additionally, the vascular endothelium influences the coagulation and fibrinolysis processes. Disruption of these processes underpins the mechanism of atherosclerosis of the blood vessels [69,70,72,73,74,75]

However, the process begins with the structure of the endothelium and the function of its cells. They produce a series of different cytokines and chemokines like C-X-C motif chemokine 12 (CXCL12), which initiates inflammation and the recruitment of cells which attend to this process. Endothelial cell receptors such as C-X-C chemokine receptor type 4 (CXCR4) and atypical chemokine receptor 3 (ACKR3) can be introduced as new targets for the treatment of atherosclerosis. However, the role of arterial ACKR3 is still not fully known, especially in reference to the human system [74]. In terms of structural considerations, endothelial glycocalyx assumes a pivotal role. It exerts a notable influence on, for instance, anticoagulant mechanisms. It is a structure consisting of proteoglycans and extracellular matrix components, the function of which may be reduced or lost during the inflammatory process [75,76,77].

Both vasoconstrictors and vasodilators play a significant role in the proper functioning of the cardiovascular system [78,79].

In addition to nitrogen oxide (NO), molecules that stimulate vasodilation include hydrogen sulfide, carbon monoxide, arachidonic acid metabolites, and hydroperoxide. The endothelium also produces opposing compounds (i.e., vasoconstrictors such as endothelin 1, angiotensin II, thromboxane A2, thrombin, and superoxide anion). These substances are essential in maintaining vascular homeostasis [69,80,81].

Endothelial dysfunction occurs under the influence of many factors, including local hemodynamic changes. Endothelial dysfunction may be considered a change in endothelial phenotype, which significantly affects, inter alia, the regulation of homeostasis, coagulation processes, fibrinolysis, or vascular tone. The epithelium is more susceptible to damage during turbulent blood flow and increased shear stress gradients. Sites such as vascular bifurcations or branches will therefore be more vulnerable [82]. When the heart pumps blood, it causes it to flow through the blood vessels in a pulsatile manner. Damage during this flow is influenced by the frictional force of the blood components, hydrostatic pressure, vessel wall stress, and cyclic stress deformation [52]. Furthermore, arterial stiffness is a significant risk factor for vascular atherosclerosis. Excessively hardened vessel walls impact the intercellular junction of the endothelium, leading to endothelial leakage and localized inflammation. Increasing evidence indicates that reducing intercellular connections results in excessive endothelial permeability. Studies in mice demonstrated that treatment with lysyl oxidase inhibitor can decrease arterial stiffness and atherosclerosis development [83]. Abnormally increased endothelial permeability leads to subendothelial lipid accumulation, which predisposes the formation of an inflammatory reaction involving leukocytes and further endothelial remodeling [84].

**Figure 2:**
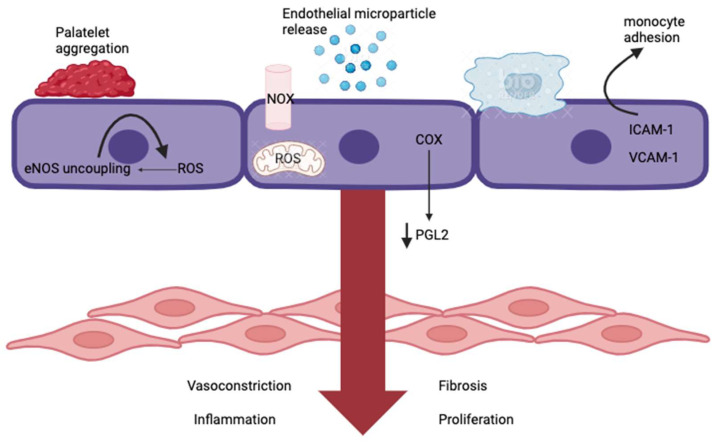
Physiopathology of endothelial dysfunction, with significant involvement of ROS in atherosclerosis pathogenesis. The arrow indicates the consequences resulting from vascular endothelial damage, which leads to localized inflammation, fibrosis, proliferation, and vasoconstriction. Here, eNOS = endothelial nitric oxide synthase; ROS = reactive oxygen species; NOX = nitrogen oxides; COX = cyclooxygenase; PGL2 = prostaglandin 2; ICAM-1 = intracellular cell adhesion molecule 1; and VCAM-1 = vascular cell adhesion molecule 1.

For vascular endothelial dysfunction in the context of atherosclerosis, we should mainly consider the activation of the endothelium. If the right conditions are present, such as the previously mentioned hemodynamics of blood flow being altered, and the endothelium is damaged, then molecular activation takes place in the form of expression of chemokines, cytokines, and adhesion molecules that will interact with leukocytes, platelets, and other immune cells [85]. Under the influence of cardiovascular risk factors, exposure leads to an elevated production of ROS within endothelial cells, leading to endothelial dysfunction through the activation of prothrombotic and proinflammatory pathways, resulting in lipid peroxidation, protein oxidation, and nucleic acid oxidation as depicted in Figure 2. Notably, mitochondrial DNA is more susceptible to ROS-induced damage compared with nuclear DNA. As a result of this damage, disturbances in the mitochondrial physiology and ATP synthesis depletion occur, leading to increased ROS formation and apoptosis. Research findings demonstrate that the accumulation of mitochondrial ROS in endothelial cells accelerates the development of atherosclerotic plaque in mice subjected to a high-fat diet. This phenomenon triggers pyroptosis within these cells, a form of programmed cell death that significantly contributes to the progression of atherosclerosis [86]. New therapeutic targets are focusing on the mitigation of a mitochondrial impact by ROS, induction of mitochondrial biogenesis, or targeted delivery of antioxidants to specific sites. Additionally, diminishing endogenous oxidation production within mitochondria can also serve as a preventative measure against atherosclerosis. The significant interrelation between mitochondrial function and atherosclerosis unveils novel therapeutic strategies, yet further investigations are imperative to chart the optimal course forward [87].

A characteristic feature of the initial stages of atherosclerosis is the local accumulation of monocytes and lymphocytes in the dysfunctional inner membrane of the vessel wall. For this to occur on the surface of this membrane, adhesion molecules involved in the interactions of vessel wall cells with blood cells appear. Adhesion molecules in the endothelium include E-selection, P-selection, intercellular adhesion molecule-1, or VCAM-1. Their recruitment under appropriate stimuli leads to accumulation of the previously mentioned monocytes and lymphocytes [37,83].

Recruited monocytes differentiate into macrophages and transform into foam cells through the uptake of extracellularly modified lipoproteins [88]. Activated chemokines, cytokines, and growth factors also induce the process of fibromuscular plaque formation. This creates a fibrous cap covering the previously formed core of the atherosclerotic plaque [89].

Currently, efforts are being made to identify novel therapeutic targets for atherosclerosis treatment. Many of these targets revolve around vascular endothelial damage, as it constitutes an essential part of its pathogenesis. Activation of purinergic signaling is among the emerging therapeutic avenues. ATP is released by endothelial cells in response to shear stress, exerting its effects through receptors such as P2X4, P2X7, and P2Y2, thereby modulating the vascular tone. Purinergic activation, mediated by A2A, P2X4, P2X7, and P2Y1 receptors, participates in inflammatory responses and leukocyte adhesion to endothelial cells. Further research is warranted to discern the actions of individual P2 receptors, rendering them effective therapeutic targets [90]. Recent investigations have also unveiled strategies involving the blockade of KCa3.1 channels for treatment. This approach inhibits smooth muscle cell proliferation and reduces ROS secretion within blood vessels. However, these channels are also present in immune cells, leading to the non-selective effects of potential blockers [91]. Aside from that, attention is also directed toward vascular endothelial damage caused by hyperhomocysteinemia now. Homocysteine induces inflammation, and apoptosis elevates oxidative stress and disrupts NO production. Lowering homocysteine levels in the human body may serve as another therapeutic target for atherosclerosis [92].

## 5. Uric Acid

A serum UA level higher than 6.8 mg/dL is considered to be an abnormally high level of UA [93]. Hyperuricemia is the fourth most important risk factor for atherosclerosis after hypertension, diabetes, and hyperlipidemia [94,95]. Rising evidence from epidemiological and genetic data suggests that a high UA level is significant to the occurrence and progression of atherosclerotic CVDs [96,97]. Men’s and women’s risk of death from atherosclerotic CVD increases by 48% and 126%, respectively, with every 1 mg/dl increase in serum UA levels [98]. According to the existing data, UA promotes the onset of atherosclerosis by interfering with lipid metabolism, decreasing endothelial cell production of nitric oxide, triggering the development of VSMCs, and exacerbating inflammation [99]. Moreover, UA has been linked to an increase in carotid intima-media thickness and coronary artery calcium [99]. This chapter will focus on the role of UA in the pathogenesis of atherosclerosis.

First, morphological plaque characteristics appear to differ among patients with high UA levels. In accordance with the analysis which examined the morphological features of atherosclerotic plaques, in patients with high UA levels, the plaque had a wider maximum lipid arc, longer calcification length, and thinner minimum fibrous cap thickness. According to a correlation analysis, the UA value was inversely associated with the minimum fibrous cap thickness and positively associated with the maximum lipid arc, average lipid arc, and calcification length [100]. Additionally, Kramer et al. showed that reducing the UA levels prevented the development of atherosclerotic plaque in mice, which were treated with uricase gene transfer and xanthine oxidase inhibitors to lower the UA levels [101].

Oxidative stress, inflammation, and endothelial dysfunction are the pathologic processes linked to higher serum UA levels. Therefore, it is not unexpected that elevated serum UA is linked to various negative consequences, such as hypertension, chronic kidney diseases, and CVDs [102].

A series of recent studies discussed the relationship between the renin–angiotensin–aldosterone system (RAAS) and UA, together with their role in atherosclerosis. Angiotensin II (AngII), a component of the RAAS, affects multiple cellular processes and can cause vasoconstriction, regulate acid-base balance, modulate immune and inflammatory pathways, and stimulate the release of chemokines. UA has been found to activate the RAAS and promote the expression of angiotensinogen, angiotensin-converting enzyme, and AngII receptors, as well as stimulate oxidative stress, inflammation, and smooth muscle cell proliferation. Additionally, UA and AngII together may lead to a more complex inflammatory and oxidative response compared with their individual effects [102,103,104,105,106,107].

These studies discovered that UA might increase the amount of inflammatory components and intensify intercellular adhesion molecule formation, which would exacerbate inflammatory responses and accelerate the development of atherosclerosis [108,109,110]. Subendothelial macrophages transform into foam cells in response to oxidative stress due to high UA and xanthine oxidase (XO) levels. The increased XO activity leads to the generation of ROS, resulting in oxidative stress, inflammation, and damage to the vascular endothelium. These processes contribute to the progression of arteriosclerosis and the formation of plaques, potentially increasing the risk of CVDs. Japanese population studies have suggested that plasma XO activity could serve as a reliable biomarker for CVDs. Furthermore, XO activity and subsequent production of UA and ROS have been implicated in promoting the early stages of CKD by affecting microcirculation, causing tissue damage, and contributing to micro-artery dysfunction and hypertension [111,112]. Furthermore, the foam cells enhance the production of inflammatory cytokines, therefore aggravating the inflammation. Moreover, it is also acknowledged that intracellular UA contributes to the development of oxidative stress. The development of atherosclerosis is accelerated by oxidative stress, which stimulates hyperuricemia [79]. Primarily, the enhanced activity of xanthine oxidase during the metabolism of UA results in the generation of ROS [113]. Arteriolar smooth muscle cells migrate, proliferate, and produce monocyte chemotactic protein-1 once the ROS is produced. The development of foam cells is aided by xanthine oxidase [114]. Furthermore, UA stimulates nicotinamide adenine dinucleotide phosphate (NADPH) oxidase, thus resulting in a reduction in NO bioavailability and an increase in protein nitrosylation and lipid oxidation [115]. Overproduction of ROS negatively affects NO accessibility, although UA also limits NO synthesis. Furthermore, increased ROS production and NADPH oxidase activation lead to mitochondrial damage, such as reduced mitochondria and adenosine triphosphate production [116,117]. Yan et al. established that heart diastolic failure, cellular apoptosis, interstitial fibrosis, and elevated calpain-1 and endoplasmic reticulum stress were all promoted by the presence of hyperuricemia in rat models [118]. The studies presented in this section are summarized in Table 1.

Interestingly, Hu et al. investigated the effect of HDL-C on carotid atherosclerosis at different serum UA levels. Elevations in the UA levels were associated with HDL-C dysfunction [119].

Yu et al. established that a high level of UA promotes atherosclerosis by targeting nuclear factor erythroid 2-related factor 2 (NRF2)-mediated autophagy dysfunction and ferroptosis. Ferroptosis is a recently identified type of controlled cell death which is primarily driven by iron-dependent lipid peroxidation and has played a role in several pathogenic diseases such as CVDs, cancer development, and acute kidney disease. A high UA level potentially accelerates the development of atherosclerosis by influencing the ferroptosis of the foam cells. Moreover, a high UA level inhibits NRF2, which is a key antioxidant regulator critical to supporting metabolic and redox balance by controlling cellular antioxidants, thus promoting the formation of atherosclerotic plaques [120].

**Figure 3:**
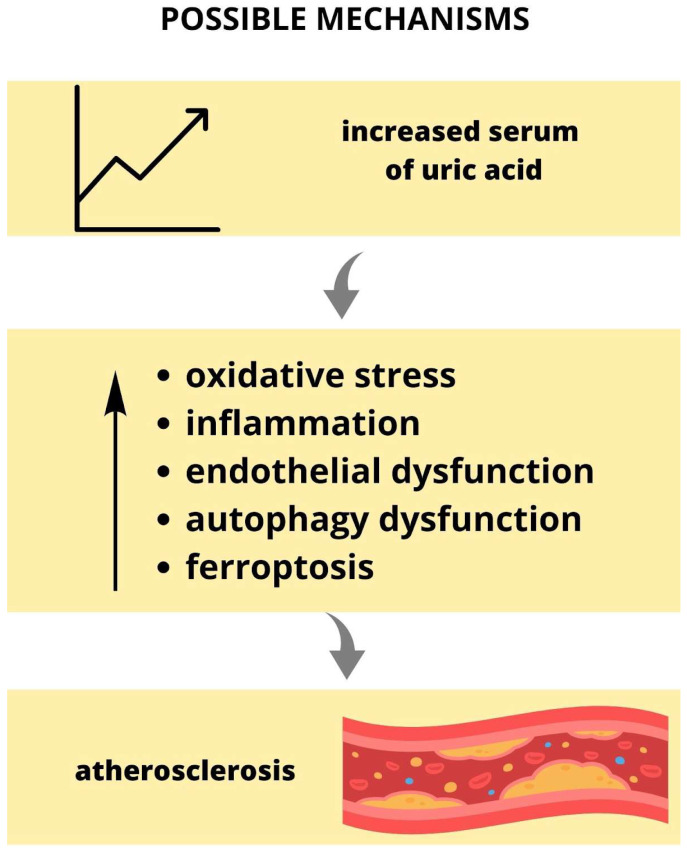
Increased UA serum level is associated with the development and progression of atherosclerosis. UA = uric acid.

In conclusion, UA exerts a pro-atherogenic effect on plaque development (Figure 3). UA enhances oxidative stress and destabilizes NO, contributing to vasoconstriction and endothelial dysfunction. Mitochondrial injury due to oxidative stress results in destabilization of the plaques. UA-induced inflammation via various inflammatory signaling pathways contributes to the progression of atherosclerosis.

These findings might contribute to a deeper understanding of the role of UA in the pathogenesis of atherosclerosis. The potential of lowering the UA levels in patients with hyperuricemia and CVDs could result in a better clinical treatment strategy.

## 6. Vitamin D

### 6.1. Vitamin D and Its Metabolism

Vitamin D plays an important role in the human body. The first studies on vit. D and its impact on the human body concerned rickets and date back to the turn of the 20th century. They led to the identification of a deficiency of vit. D as the etiology of this disease [121]. Vit. D deficiency is the most common nutritional deficiency, estimated to affect one billion people worldwide [122]. Vit. D is a secosteroid derived from cholesterol in animals (cholecalciferol, or vitamin D3) and ergosterol in plants (ergocalciferol, or vitamin D2). Despite the results of previous studies suggesting that both forms of the vitamin are equally effective at increasing the total and free serum 25-hydroxyvitamin D (25OHD) levels in humans, some recent studies suggested that the D3 form has a more significant contribution [123,124,125]. Vit. D metabolism is a complex process involving many organs. Vit. D can be synthesized de novo in the skin through exposure to ultraviolet B light, which converts 7-dehydrocholesterol to cholecalciferol. It can also be absorbed by ingestion. The further fate of cholecalciferol includes its brief binding to a vitamin D-binding protein and transport to the liver, where the liver enzyme 25-hydroxylase (CYP2R1) converts vit. D into 25OHD. The next stage of hydroxylation takes place in the kidneys, where the active form—1,25(OH)2D—is formed, which is then released into the bloodstream and binds to the vit. D-binding protein [126].

Vit. D needs to be activated by 25-hydroxylation in the liver, yielding 25OHD, and through 1α-hydroxylation in the kidney yielding the vit. D hormone 1,25(OH)2D. It is now believed that almost all of the biological effects of vit. D are mediated by its active form, 1,25(OH)2D, signaling mainly through the intracellular vit. D receptor (VDR) [127]. As a lipophilic hormone, 1,25(OH)2D can penetrate the cell membrane and bind to the VDR present in the cytoplasm or nucleus of the target cells. VDR is a ligand-activated transcription factor and regulates gene expression. More than 30 cell types are known to express VDR, suggesting multiple roles for vit. D beyond mineral homeostasis [128].

Vit. D metabolism is highly regulated by levels of calcium, phosphate, fibroblast growth factor 23, and parathyroid hormone [129].

### 6.2. Vitamin D and Its Impact on Atherosclerosis

The level of 25(OH)D is influenced by the method of ingesting and the source of foods rich in vit. D. It is also dependent on the exposure to sunlight and fresh air during physical activities. Race and ethnicity also influence differences in the markers of vit. D metabolism [130]. However, there is currently no consensus on determining optimal serum levels and dietary requirements. Additionally, the sufficiency threshold may vary for different diseases and conditions, making it difficult to establish optimal reference values [131,132]. For an exceptionally long time, it was believed that the only physiological role of vit. D was to regulate calcium and phosphate metabolism. However, research by Scragg et al. scored seasonality in patients suffering from CVDs and attributed it to low levels of 25OHD the patients exhibited during the winter, which could be considered a groundbreaking finding pointing toward a further link between vit. D and diseases of the cardiovascular system [133].

Over the years, numerous publications have included both case studies and experimental findings that have linked vit. D levels to CVD risk in its origin and occurrence. As a result, further research into vit. D supplementation for the clinical improvement of patients has been conducted. However, some studies did not confirm this correlation and did not prove the effectiveness of such treatment [134].

The focus of this article concerns atherosclerosis and innovative approaches to treating it by means of research on new therapeutic solutions and pathophysiological factors. Due to the complexity of this condition, interactions between environmental and genetic factors are still being studied.

Recent experimental and clinical studies showed evidence for the effect of vit. D signaling on the modulation of atherosclerosis pathogenesis. Vit. D signaling reduces the expression of TNFα, IL-6, IL-1, and IL-8 in isolated blood monocytes. IL-6 suppression leads to a decrease in the synthesis of acute phase C-reactive protein. This may contribute to the formation of atherosclerotic changes [135,136]. Vit. D deficiency in a pig study showed increased activation of nuclear factor κB, indirectly supporting the anti-inflammatory role of vit. D [137]. Vit. D deficiency increases the adhesion molecules of vascular cells and E-selectin, which play a role in the formation of atherosclerotic plaques. However, it has been confirmed that supplementation in deficient patients reduces total cholesterol, triglycerides, and LDL cholesterol and increases HDL cholesterol. It also affects the vascular tone by modifying endothelial nitric oxide production. Deficiency can lead to oxidative stress which, by intensifying inflammation, significantly affects development of the atherosclerotic process in the intima [138]. It has been found that vit. D deficiency can cause endothelial damage in the early stages of atherosclerosis without developing clinical symptoms of CVD [139].

A link has been found between vit. D deficiency or supplementation and the development of a myocardial infarction or a stroke. Lower levels of vit. D are associated with a higher risk of CVD and the development of hypertension. Unfortunately, conclusions regarding the relief of symptoms in CVDs are still unclear and may, with further research, counter many prejudices [140]. Using computed tomography angiography, a study was conducted in which it was found that people with lower levels of vit. D than those considered sufficient were slightly more likely to develop coronary atherosclerosis [141]. Severe vit. D deficiency has also been listed as a significant risk factor for ischemic stroke [142,143]. There is also evidence that lower concentrations of 25(OH)D in the serum of postmenopausal women are associated with significant narrowing of the coronary arteries [144].

Since obesity accelerates the aging process of cells, its relationship with vit. D deficiency should also be carefully considered. One study conducted on this topic showed that obesity may be an important pathogenetic factor in subclinical atherosclerosis, and vit. D supplementation may have a protective role against the occurrence of comorbidities [145].

On the other hand, vit. D supplementation has not been shown to reduce the CVD burden in older adults. A 2020 randomized controlled trial (RCT) found that vit. D supplementation at 2000 IU/day for 3 years did not reduce elevated systolic and diastolic blood pressure, which is often increased due to atherosclerotic disease. This suggests that vit. D supplementation for less than 3 years does not influence SBP or DBP. Elevated arterial blood pressure is a known cause of atherosclerosis, and high blood pressure is a risk factor for developing atherosclerosis [146]. A 2017 RCT found no beneficial influence of vit. D in treating CVD, including the direct consequences of systemic and coronary atherosclerosis such as hypertension or angina [147]. The Randomized Evaluation of Calcium or Vit. D (RECORD) trial showed no effect from low-dose vit. D supplementation (400–800 IU) on 2–7-year cardiovascular mortality and all-cause mortality [148]. The Women’s Health Initiative trial showed non-significant deleterious effects of vit. D and calcium supplementation on non-fatal myocardial infarction, death from coronary artery disease, and the need for vascularization. However, the results also support the possibility that these supplements may reduce mortality in postmenopausal women [149]. A study of 26,000 patients found that a daily dose of 2000 IU of vit. D combined with 1 g of omega-3 fatty acids did not reduce cardiovascular events [150]. Further research is needed to definitively evaluate the role of vit. D supplementation in reducing myocardial infarction, stroke, or overall CVD burden in deficient or insufficient patients.

## 7. miRNA Expression

MicroRNAs (miRNAs) are small non-coding RNAs that have diverse cellular roles [151] but are best known for silencing and fine-tuning the expression of messenger RNA (mRNA) transcripts. They primarily affect gene expression levels via targeting mRNA, and their relative levels have a major role in carcinogenesis and other diseases [152]. The first-known role of miRNAs is to influence mRNA via recognition sites in the 3′untranslated region, which regulates its stability [153]. Recently, several discoveries have been made that show a significant relationship between miRNAs and atherosclerosis.

By regulating the expression of genes that are important in the pathogenesis of processes leading to atherosclerosis, miRNAs can affect the level of synthesized protein in cells. This protein may be significant in dysregulation, which then contributes to the intensification of atherosclerotic processes [154], and miRNAs are also very important in NO production and endothelial growth [155], which could be illustrated with the following example. MiR-217 was strongly stimulated during the aging of human endothelial cells, resulting in the downregulation of silent information regulator (Sirt1) expression. In these cells, Sirt1 activates endothelial NO synthase (eNOS), which generates NO [156]. NO participates in regulation of the vascular tone, cell proliferation, leukocyte adhesion, and platelet aggregation. De Yébenes V.G. et al., in their research, also emphasized that miR-217 inhibitors have a significant therapeutic potential in CVDs, including atherosclerosis associated with the aging process. Further analysis revealed that human plasma miR-217 is a biomarker of vascular aging and cardiovascular risk [157]. Moreover, circulating levels of miRNAs enriched in vascular cells in patients can serve as a marker of disease severity and phenotypes [158].

NM-Exos helps promote vascular smooth muscle migration and proliferation, and miR-21-3p plays a key role in regulating the NM-Exos-induced effects. Therefore, exosomal miR-21-3p from nicotine-treated macrophages can accelerate the development of atherosclerosis by supporting this relation [159]. Sharma A.R. et al. discussed various circulating miRNAs such as miR-17, miR-17-5p, miR-29b, miR-30, miR-92a, miR-126, miR-143, miR-145, miR-146a, miR-212, miR-218, miR-221, miR-222, and miR-361-5p as biomarkers in the clinical diagnosis of atherosclerosis. The authors hope that their work will allow doctors to use better treatments and diagnostics in this area [160].

Liu X. et al. demonstrated that the NFATc3 macrophage upregulates miR-204 to reduce the SR-A and CD36 levels, thereby preventing foam cell formation and atherosclerosis. The NFATc3/miR-204 axis may be another target in the development of individual therapy against atherosclerosis [161]. MiR-320b has been recognized as a novel modulator of cholesterol efflux from macrophages [162]. The miR-33 family includes essential regulators of cellular lipid homeostasis and lipoprotein metabolism, participating in ABCA1 and ABCG110-16 regulation. Defects in miR-33 affect the development of atherosclerosis due to its protective effect on macrophages. Inhibition of miR-33 by pH low-insertion-peptide (pHLIP)-driven targeting improves atherosclerotic regression because it decreases lipid accumulation [163]. Therefore, it is rational to make use of pHLIPs in the treatment of advanced atherosclerosis through pharmacological inhibition of miR-33 in macrophages [156].

DNA methylation is a key epigenetic modification in regulating cell function by silencing the relative gene expression. DNA and miRNA methylation are important epigenetic factors in AS. This study reviews recent insights into the role of miRNA and DNA methylation and their interaction in AS progression [164]. DNA methylation in mammals appears at the fifth position of cytosine (5mC) in CpG dinucleotide and participates in transcriptional repression and cellular regulation. While the addition of methyl groups could be accomplished by DNA-methyltransferase, these can be removed passively (DNA replication) and actively through sequential oxidation [165]. DNA and RNA methylations are regulated by multiple stimuli, which are considered risk factors in atherosclerosis, such as oxidative stress and hyperglycemia. Finally, it is worth mentioning that recent studies have shown that both DNA and miRNA methylation are principal factors involved in the pathogenesis of atherosclerosis [166]. The genes presented in this section are summarized in Table 2.

## 8. Conclusions

Atherosclerosis is a common vascular aging condition. Nevertheless, a variety of factors could have an impact on this process, leading to an increase in cardiovascular risk and a high rate of morbidity and mortality.

In this review, we focused on the important molecular aspects of arteriosclerosis. We paid attention to the role of the immune system, its dysfunctions, and its influence on the development of this disease. Endothelial dysfunction, its pathomechanism, and its influence on the development of CVDs were also considered to be of interest. Moreover, as advancing age is a significant risk factor for atherosclerotic CVD, we discussed the vascular intrinsic and extrinsic mechanisms of how aging influences the pathology of atherosclerosis. Furthermore, we drew attention to the influence of several factors, such as UA, vit. D, and miRNA, since their key role in the pathogenesis of atherosclerosis has been established in research in recent years.

**Figure 4:**
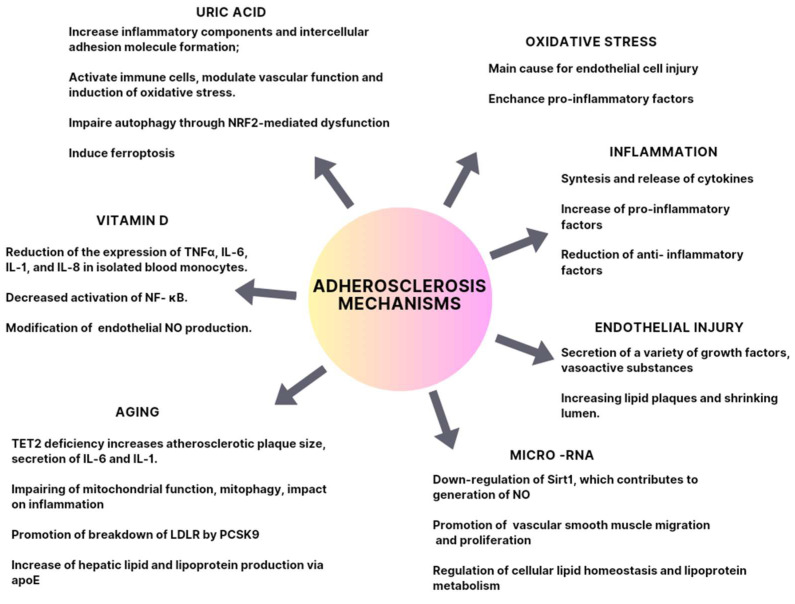
Association of oxidative stress, inflammation, aging endothelial injury, uric acid, vitamin D, and micro-RNA with atherosclerosis.

These findings might shed new light on the cellular mechanisms of atherosclerosis and prospective targets for prevention and treatment in the future. The factors presented in this review are summarized in Figure 4.

However, the significant scientific achievements of recent years and the many new discoveries and mechanisms still require careful attention and additional studies.

## Figures and Tables

**Table 1:** Association of UA with inflammation.

Study	Year	Study Design	Findings
Polito et al. [110]	2021	Review article	XO plays a significant role in CVDs, including atherosclerosis, through various mechanisms such as oxidative stress, inflammation, endothelial dysfunction, and modulation of purinergic signaling.
Kotozaki et al. [111]	2023	Population-based study	Higher XO activity in human plasma is associated with an increased risk of CVDs, indicating its potential role as a biomarker and a therapeutic target.
Kimura et al. [112]	2021	Review article	UA contributes to the pathogenesis of atherosclerosis by promoting inflammation through the activation of immune cells, modulation of vascular function, and induction of oxidative stress.
Yan et al. [117]	2018	In vitro study (cardiomyocytes)	UA induces cardiomyocyte apoptosis by activating calpain-1 and endoplasmic reticulum stress, suggesting a potential mechanism by which hyperuricemia contributes to cardiac dysfunction.
Hu et al. [118]	2022	Clinical study	Elevated serum US levels are associated with a pro-inflammatory state and have an impact on the role of HDL-C in promoting carotid atherosclerosis, highlighting the link between UA, inflammation, and atherosclerosis.
Yu et al. [119]	2022	Experimental study (cellular and animal models)	High levels of UA promote atherosclerosis by impairing autophagy through NRF2-mediated dysfunction and inducing ferroptosis, providing insights into the molecular mechanisms underlying the pro-atherogenic effects of UA.

XO = xanthine oxidoreductase-; CVDs = cardiovascular diseases; UA = uric acid; and HDL-C = high-density lipoprotein cholesterol.

**Table 2:** Selected miRNAs unregulated in atherosclerosis or involved in signaling pathways in the development of atherosclerosis.

Gene	Cell/Tissue Type	Function or Association with Disease
miR-1	blood, VSMCs	Expression is associated with subclinical atherosclerosis. Induces VSMC differentiation.
miR-10a	serum, VSMCs	Negative regulator of SMC differentiation.
miR-19a	ECs, B cells, VSMCs	Promotion of VSMC proliferation. Suppression of IL-10-mediated immunomodulation. Mediates the effects of laminar flow and cell cycle progression.
miR-33a/b	Cells from liver, macrophages, fibroblasts	Regulation of cholesterol homeostasis.
miR-146a	VSMCs, EPCs	VSMC proliferation. Neointimal hyperplasia.
miR-302a	macrophages	Regulation of cholesterol efflux.
miR-221 and miR-222	VSMCs	VSMC proliferation. Neointimal hyperplasia.

miR = miRNA; VSMC = vascular smooth muscle cell; SMC = smooth muscle cell; EC = endothelial cells; and EPC = endothelial progenitor cell.

## Data Availability

Not applicable.

## Abbreviations

CVD	cardiovascular disease
UA	uric acid
Vit. D	vitamin D
1,25(OH)2D	calcitriol, 1,25-dihydroxyvitamin D
miRNA	microRNA
ACS	acute coronary syndrome
CKD	chronic kidney disease
LDL	low-density lipoproteins
CVDs	cardiovascular diseases
CCs	cholesterol-soluble compounds
NETs	neutrophil extracellular traps
Tet2	ten-eleven translocation 2
HDL	high-density lipoprotein
oxLDLs	oxidized low-density lipoproteins
ROS	reactive oxygen species
TNFα	tumor necrosis factor α
VCAM-1	vascular cell adhesion molecule-1
RNS	reactive nitrogen species
LDL-C	LDL-cholesterol
CHIP	clonal hematopoiesis of indeterminate potential
VSMCs	vascular smooth muscle cells
PCSK9	proprotein convertase subtilisin/kexin type 9
LDLRs	low-density lipoprotein receptors
AAV	adeno-associated virus
PCL	partial carotid ligation
ACKR3	atypical chemokine receptor 3
NO	nitrogen oxide
RAAS	renin–angiotensin–aldosterone system
AngII	angiotensin II
XO	xanthine oxidase
NADPH	nicotinamide adenine dinucleotide phosphate
NRF2	nuclear factor erythroid 2-related factor 2
25OHD	25-hydroxyvitamin D
VDR	intracellular vitamin D receptor
RCT	randomized controlled trial
miRNAs	microRNAs
MRN A	messenger RNA
Sirt1	silent information regulator
pHLIP	pH low-insertion peptides
